# Editorial: Early diagnosis and mechanism analysis of non-motor symptoms of Parkinson's syndrome

**DOI:** 10.3389/fneur.2024.1434924

**Published:** 2024-06-12

**Authors:** Min Yuan, Qian Xu, Mei Han, Haili Pan

**Affiliations:** ^1^Department of Neurology, Jiangxi Provincial People's Hospital, The First Affiliated Hospital of Nanchang Medical College, Nanchang, China; ^2^Department of Neurology, Xiangya Hospital, Central South University, Changsha, China; ^3^Department of Genetic Medicine, School of Medicine, Johns Hopkins University, Baltimore, MD, United States; ^4^Neurological Institute of Jiangxi Province and Department of Neurology, Jiangxi Provincial People's Hospital, The First Affiliated Hospital of Nanchang Medical College, Nanchang, China

**Keywords:** non-motor symptoms, early diagnosis, Parkinson's syndrome, neuropsychiatric symptoms, autonomic dysfunction

## Introduction

It is with great pleasure that we introduce this Research Topic of Frontiers in Neurology, dedicated to the theme “*Early diagnosis and mechanism analysis of non-motor symptoms of Parkinson's syndrome*.” This Research Topic features eight insightful articles that explore various aspects of non-motor symptoms in Parkinson's syndrome, including neuropsychiatric symptoms, autonomic dysfunctions, cognitive impairments, and their associations with age and gender.

Non-motor symptoms of Parkinson's syndrome have garnered increasing attention in recent years due to their profound impact on patients' quality of life. These symptoms often manifest early in the disease course and can serve as valuable indicators for early diagnosis (1). Despite their significance, non-motor symptoms are frequently underrecognized and undertreated in clinical practice. This Research Topic seeks to address this gap by presenting cutting-edge research and novel insights into the early identification, underlying mechanisms, and management strategies for non-motor symptoms in Parkinson's syndrome.

Parkinson's syndrome encompasses a range of clinical conditions caused by various etiologies, mainly including primary Parkinson's disease, Parkinson's superposition syndrome, secondary Parkinson's syndrome, and hereditary Parkinson's syndrome (2). Each of these conditions presents with a unique constellation of symptoms and disease progression patterns. While motor symptoms such as bradykinesia, rigidity, resting tremor, and postural instability are hallmark features, non-motor symptoms are equally prevalent and significantly impact the quality of life of patients. Importantly, these non-motor symptoms often precede motor symptoms, potentially serving as sensitive predictors of Parkinson's syndrome (3). However, the early diagnosis and underlying mechanisms of these non-motor symptoms remain largely unknown, resulting in diagnostic delays and suboptimal treatment.

## Highlights from the contributions

### Minor hallucinations in Parkinson's disease

Wang Y. et al., in their study “*Clinical features of minor hallucinations in different phenotypes of Parkinson's disease: a cross-sectional study*,” delve into the clinical features of minor hallucinations across various PD phenotypes. Their cross-sectional study provides valuable insights into the prevalence and manifestation of these hallucinations, highlighting differences among PD subtypes. These findings pave the way for early identification and personalized treatment strategies for managing hallucinations, which are often overlooked but significantly affect the patients' daily lives and quality of life.

### Predicting fatigue using gait analysis

Wang H. et al. present an innovative approach in “*Predicting the fatigue in Parkinson's disease using inertial sensor gait data and clinical characteristics*.” By leveraging advanced inertial sensor technology and gait analysis, they propose a novel method for predicting fatigue in PD patients. This study underscores the potential of technology to enhance the accuracy of diagnosing non-motor symptoms, offering a non-invasive and effective tool for clinicians. Early identification of fatigue can lead to timely interventions that may help mitigate this debilitating symptom, thereby improving patients' overall functioning and quality of life.

### Constipation in multiple system atrophy

Chen et al.'s study, “*Constipation in multiple system atrophy: a pilot study in Chinese patients*,” focuses on the prevalence and severity of constipation in MSA patients within a Chinese cohort. This research emphasizes the importance of understanding autonomic dysfunctions in different geographical and ethnic contexts, highlighting the need for tailored treatment approaches. Constipation is a common but often underreported symptom that can significantly impact patients' comfort and wellbeing, and this study brings attention to its management in a specific patient population.

### Theory of mind deficits and dopaminergic medication

Usnich et al. explores cognitive impairments in her work, “*Theory of mind deficits in Parkinson's disease are not modulated by dopaminergic medication*,” complemented by a corrigendum. The study reveals limited influence of dopaminergic medications on Theory of Mind (ToM) deficits, suggesting that non-pharmacological interventions might be necessary. This finding opens avenues for diverse therapeutic approaches to manage cognitive symptoms in PD, emphasizing the need for comprehensive care strategies that address both motor and non-motor symptoms to improve overall patient outcomes.

### Urinary dysfunction and extracellular vesicles

In “*Urinary extracellular vesicle dynamics in Parkinson's disease patients with urinary dysfunction*,” Roy et al. investigates the mechanisms behind urinary dysfunction in PD. By analyzing urinary extracellular vesicle dynamics, the study identifies potential biomarkers that could aid in diagnosis and treatment, offering new directions for clinical practice. Urinary dysfunction is a distressing symptom that can significantly impair quality of life, and identifying biomarkers for early diagnosis and monitoring can lead to better management strategies.

### Age and gender differences in non-motor symptoms

Maas et al. examines the influence of age and gender in “*Age and gender differences in non-motor symptoms in people with Parkinson's disease*.” This research highlights significant variations in non-motor symptom presentation, underscoring the necessity of considering individual differences in clinical settings to develop personalized treatment plans. Understanding how age and gender influence symptomatology can help clinicians provide more tailored and effective care, addressing the unique needs of different patient groups.

### Online cognitive testing

Binoy et al.'s article, “*Online cognitive testing in Parkinson's disease: advantages and challenges*,” evaluates the use of online cognitive tests for PD patients. It discusses the advantages, such as accessibility and convenience, as well as challenges like environmental control and data accuracy. This study provides valuable insights into the role of remote tools in early diagnosis and ongoing assessment, especially in the context of telemedicine. As healthcare increasingly moves toward digital solutions, understanding the potential and limitations of online cognitive testing is crucial for integrating these tools into routine clinical practice.

## Future directions

The diverse range of studies presented in this Research Topic not only expands our understanding of non-motor symptoms in Parkinson's syndrome but also offers new perspectives for early diagnosis and intervention. These findings will help us better comprehend the underlying mechanisms of non-motor symptoms, identify novel diagnostic biomarkers, and explore innovative treatment targets. Ultimately, these advancements aim to improve the quality of life for patients with Parkinson's syndrome.

We extend our deepest gratitude to all the authors, reviewers, and the editorial team for their hard work and contributions. We hope this Research Topic will inspire further research and discussions on non-motor symptoms in Parkinson's syndrome, driving progress in this critical area of neurology.

## Author contributions

MY: Conceptualization, Data curation, Validation, Writing – original draft, Writing – review & editing. QX: Writing – review & editing. MH: Writing – review & editing. HP: Writing – review & editing.
